# Mycosynthesis of ZnO Nanoparticles Using *Trichoderma* spp. Isolated from Rhizosphere Soils and Its Synergistic Antibacterial Effect against *Xanthomonas oryzae* pv. *oryzae*

**DOI:** 10.3390/jof6030181

**Published:** 2020-09-20

**Authors:** Balagangadharaswamy Shobha, Thimappa Ramachandrappa Lakshmeesha, Mohammad Azam Ansari, Ahmad Almatroudi, Mohammad A. Alzohairy, Sumanth Basavaraju, Ramesha Alurappa, Siddapura Ramachandrappa Niranjana, Srinivas Chowdappa

**Affiliations:** 1 Department of Microbiology and Biotechnology, Bangalore University, Jnana Bharathi Campus, Bengaluru 560056, India; shobhahonnaganga@gmail.com (B.S.); simplesumanth007@gmail.com (S.B.); Ramesha.bio@gmail.com (R.A.); 2 Department of Epidemic Disease Research, Institute for Research and Medical Consultations (IRMC), Imam Abdulrahman Bin Faisal University, Dammam 31441, Saudi Arabia; maansari@iau.edu.sa; 3 Department of Medical Laboratories, College of Applied Medical Sciences, Qassim University, Qassim, 51431 Saudi Arabia; dr.alzohairy@gmail.com; 4 Department of Studies in Biotechnology, University of Mysore, Manasagangotri, Mysore 570006, India; niranjanasr@rediffmail.com

**Keywords:** fungal nanotechnology, *Trichoderma* spp., ZnO nanoparticles, antibacterial activity *Xanthomonas oryzae* pv. *oryzae*, co-cultivation, secondary metabolites

## Abstract

The Plant Growth Promoting Fungi (PGPF) is used as a source of biofertilizers due to their production of secondary metabolites and beneficial effects on plants. The present work is focused on the co-cultivation of *Trichoderma* spp. (*T. harzianum* (PGT4), *T. reesei* (PGT5) and *T. reesei* (PGT13)) and the production of secondary metabolites from mono and co-culture and mycosynthesis of zinc oxide nanoparticles (ZnO NPs), which were characterized by a UV visible spectrophotometer, Powder X-ray Diffraction (PXRD), Fourier Transform Infrared Spectroscopy (FTIR) and Scanning Electron Microscopy (SEM) with Energy Dispersive Spectroscopy (EDAX) and Transmission Electron Microscope (TEM) and Selected Area (Electron) Diffraction (SAED) patterns. The fungal secondary metabolite crude was extracted from the mono and co-culture of *Trichoderma* spp. And were analyzed by GC-MS, which was further subjected for antibacterial activity against *Xanthomonas oryzae* pv. *Oryzae*, the causative organism for Bacterial Leaf Blight (BLB) in rice. Our results showed that the maximum zone of inhibition was recorded from the co-culture of *Trichoderma* spp. rather than mono cultures, which indicates that co-cultivation of beneficial fungi can stimulate the synthesis of novel secondary metabolites better than in monocultures. ZnO NPs were synthesized from fungal secondary metabolites of mono cultures of Trichoderma harzianum (PGT4), Trichoderma reesei (PGT5), Trichoderma reesei (PGT13) and co-culture (PGT4 + PGT5 + PGT13). These ZnO NPs were checked for antibacterial activity against Xoo, which was found to be of a dose-dependent manner. In summary, the biosynthesized ZnO NPs and secondary metabolites from co-culture of *Trichoderma* spp. are ecofriendly and can be used as an alternative for chemical fertilizers in agriculture.

## 1. Introduction

Rice (*Oryzae sativa* L.) is one of the most vital and essential nourishment sources for half of the world’s population, it belongs to the family Poaceae, and it is the most widely cultivated food crop in the world [1]. Across the world, annually, about 40% of rice crops are lost due to biotic stresses such as insects, pathogens, pests and weeds [2]. Some of the most important of these diseases are Bacterial Leaf Blight (BLB) caused by (*Xanthomonas oryzae* pv. *oryzae*), Blast (*Magnaporthe grisea*), Sheath Blight (*Rhizoctonia solani*), Sheath Rot (*Sarocladium oryzae*) and Tungro virus [3].

Rice’s BLB is one of the most damaging causes of disease, which is caused by *Xanthomonas oryzae* pv. *Oryzae* (Xoo) [4]. This bacteria restricts annual production of rice in both tropical and temperate regions of the world [5]. In the tropics, the damage is more severe than in the temperate regions [6]. The BLB disease incidence has been recorded in various parts of Asia, USA, Africa, and northern Australia [7]. Various disease management strategies are used to minimize BLB damage, such as chemical control, host–plant resistance, crop system modification, and biological control [8]. In the 1950s, chemical management of BLB in rice fields started with the preventive application of the Bordeaux mixture, other chemicals such as phenazine oxide, tecloftalam and nickel dimethyl dithiocarbamate directly sprayed on plants [9]. Synthetic organic bactericides were also recommended, such as nickel dimethyl dithiocarbamate, phenazine noxide and dithianon phenazine [10]. Overuse of chemical substances often adversely affects the environment, farmers and consumer health [11]. Biological control is an alternative method, which is ecologically sensitive, cost-effective and sustainable in BLB management [12]. An effective means of managing plant diseases can be by using antagonistic microorganisms [13]. Interaction between plant pathogens and biocontrol agents has been extensively studied, and the use of biocontrol agents is promising in protecting some commercially valuable crops [14]. Plant growth-promoting rhizobacteria (PGPR) are the most widely studied group of plant growth-promoting bacteria (PGPB) and plant growth-promoting fungi (PGPF), which colonize root surfaces and closely adhere to the soil interface—the rhizosphere—and can also be used for plants [15,16]. Species of *Trichoderma* were identified as potentially environmentally safe biofertilizer and are non-toxigenic [17]. *Trichoderma* species are effective in agriculture as biological control agents and their frequent addition to soil leads to increased crop yields and control of soil-borne pathogens worldwide [18]. Plant growth-promoting rhizobacteria are root-colonizing, free-living bacteria with beneficial effects on crop plants which work by reducing disease incidence and increasing yields [19]. It contributes to the suppression of disease by various modes of action such as antagonism, space and nutrient rivalry and induction of systemic resistance (ISR) [20]. By eliciting induced systemic resistance, PGPR indirectly mediates biological control in a variety of plant diseases [21].

The co-culture of two or more beneficial fungi can interact, stimulate or enhance the production of secondary metabolites, which are not presented in the mono or single cultures when grown separately in in vitro conditions. The co-culture also triggers certain genes which are not activated in mono or single culture, it can also stimulate various pathways when grown together in in vitro conditions [22]. 

The introduction of nanotechnology to agricultural science seems to offer promising solutions including the release of modified fertilizers and pesticides [23]. The unique and different properties of nanoparticles such as electrical conductivity, active area, hardness and chemical reactivity can be achieved by reducing the size to nanometers [24].

Biological production of nanoparticles based on natural resources has recently attracted scientific interest. Nanoparticles synthesized using natural resources are called green synthesis or biosynthesis [25]. Nanomaterial biosynthesis has provided a common point between nanotechnology and biotechnology and has led to the development of new materials used in many fields [26]. Fungi have become one of the choices in nanotechnology because of its wide variety of advantages over the bacteria, actinomycetes, plants and other physic-chemical properties. The capability of tolerance and metal bioaccumulation in fungi has made fungi a significant branch in the biosynthesis of nanoparticles [27]. Non-toxic and safe reagents are used in the green synthesis of nanoparticles, which makes them cost effective and environmentally friendly [28].

Surface atomic arrangements influence the antibacterial properties [29]. The specific arrangements of atoms on the surface are selected inorganic oxides and work by a fine-tuning of the morphology. By modifying the conditions and by examining the morphology, synthesis of inorganic oxides can be controlled morphologically [30]. Nano-particles are an alternative method that has gained significant attention in the field of plant defense [31]. In comparison, compared with other metal-NPs, the ZnO NPs have been found to be less harmful to plants and beneficial to soil micro flora [32].

Our study is mainly focused on a biological method for the management of the BLB caused by Xoo, a rice disease using co-culture and monoculture of *Trichoderma* spp., which is eco-friendly. The studies were carried out in vitro using the biosynthesis of Zinc Oxide Nanoparticles synthesized from *Trichoderma* spp., which is a new approach in agriculture for the management of the disease. To our best knowledge, this work is reported for the first time.

## 2. Materials and Methods

### 2.1. Collection and Isolation of Bacteria from Infected Rice Leaf

The infected leaf samples were collected from different places of Karnataka and were subjected for the isolation of Xoo. The infected parts of the leaves were cut into 0.5 cm^2^, the surface was sterilized with 1% sodium hypochlorite for one minute followed by 3–4 sterile distilled water washes and was then blot dried. The sterilized leaf segments were inoculated onto an agar medium of yeast extract dextrose calcium carbonate (YDC) incubated at 28 ± 2 °C for 72 h. The plates were observed for convex, mucoid and yellow color [33].

### 2.2. Identification of Isolated Bacteria by Biochemical and Molecular Characterization

The bacterial isolates were identified based on morphological, microscopic, biochemical and molecular characterization. Biochemical tests such as gram staining, catalase, oxidase test, KOH test, gelatin liquefaction, starch hydrolysis, casein hydrolysis and pectin hydrolysis were carried out as described in [34]. Molecular characterization was carried out to identify the organism at a molecular level. Using a Chromous bacterial genomic DNA isolation kit, the genomic DNA of bacterial samples were isolated following the standard protocol. PCR amplification was carried out using universal 16s rRNA primers. The obtained sequences were deposited in an NCBI GenBank and an accession number was obtained [35]. The GenBank accession numbers are as follows: *Xanthomonas oryzae* pv. *oryzae* (MBXoo53) MF787295.1, *Xanthomonas oryzae* pv. *oryzae* (MBXoo70) MF787294.1, *Xanthomonas oryzae* pv. *oryzae* (MBXoo69) MF579736.1.

### 2.3. Collection and Isolation of Trichoderma spp. from Rhizosphere Soil

Rhizospheric soil (soil around the root zone) samples of different crops were collected from different parts of Karnataka. Five grams of rhizosphere soil was collected by uprooting the plant with the soil attached to the roots. The collected soil samples were preserved in polythene bags and stored at 4 °C until further use [36]. The collected soil samples of the rhizosphere were diluted into various concentration solutions, were well-vortexed and 0.1 mL of the supernatants were poured onto PDA medium (potato dextrose agar, with chloramphenicol antibiotics) plates and incubated at 28 °C for 7 days. Colonies that appeared on the plates were isolated and reinoculated on a new PDA petri plate. After 7 days of subculturing, single-spore colonies were obtained by incubating the plates at 28 °C, and the fungal colonies were further assayed for morphological and physiological characteristics [37]. 

### 2.4. Morphological and Molecular Characterization of Trichoderma spp.

The identification of fungi was carried out based on the cultural and microscopic properties using standard manuals [38]. By using a Chromous genomic DNA isolation kit, the genomic DNA of the microbial sample was isolated following the protocol as described by the manufacturer. PCR amplification was carried using universal 18S primers. The obtained sequences were deposited in NCBI GenBank and an accession number was obtained [39]. The GenBank accession numbers are as follows: *Trichoderma harzianum* (PGT4) MH429899.1, *Trichoderma reesei* (PGT5) MH429901.1, *Trichoderma reesei* (PGT13) MH429900.1.

### 2.5. In Vitro Screening of Plant Growth-Promoting Fungi (PGPF) Strains of Trichoderma spp. for Its Antibacterial Activity by Agar Plug Method against Xanthomonas oryzae pv. oryzae (Xoo)

The mycelial disc of *Trichoderma* spp. fungi isolated from rhizosphere soil was screened for its antibacterial activity against plant pathogenic bacterial strain *Xanthomonas oryzae* pv. *oryzae* (Xoo) isolated from the infected leaf parts of rice. The different Plant Growth-Promoting Fungi (PGPF) strains of *Trichoderma* spp. were grown on a PDA petri plate for 5–7 days at 24 ± 2 °C with alternate light and dark periods. From seven-day-old, culture the fungal discs were pierced using a sterile cork borer of 5 mm in diameter. The fungal discs were transferred to a Mueller-Hinton agar (MHA) plate which was previously swabbed with the bacteria Xoo. The MHA plates were kept for incubation at 28 °C for 24 h and after incubation, the results were observed [40].

### 2.6. Trichoderma–Trichoderma Interactions through Co-Culture

In our study, co-culture was carried out to evaluate the ability of selected *Trichoderma* spp. cultures to produce secondary metabolites. This was carried out on solid culture medium potato dextrose agar (PDA) for three selected fungi: *T. harzianum* (PGT4), *T. reesei* (PGT5) and *T. reesei* (PGT13) based on its antibacterial activity against *Xanthomonas oryzae* pv. *oryzae*. These fungi were cultured on PDA and incubated at 25 °C for 7 days. After incubation, 10 mm of fungal discs were taken from actively growing margins of *Trichoderma* spp. on PDA plates. Two different *Trichoderma* spp. cultures of 10 mm were placed on PDA plates at the opposite ends of the petri plates, making a total of 3 dual cultures (pairwise combinations). Triplicates of both dual and monocultures were made and incubated under dark conditions for 9 days at 25 °C [41].

### 2.7. Production of Secondary Metabolites from Trichoderma spp.

The agar plugs of *Trichoderma* spp., measuring 7 mm in diameter, were taken from actively growing margins of *T. harzianum* (PGT4), *T. reesei* (PGT5) and *T. reesei* (PGT13) cultures grown on PDA media and were inoculated individually: dual cultures (*T. harzianum* (PGT4) and *T. reesei* (PGT5); *T. reesei* (PGT5) and *T. reesei* (PGT13) and *T. reesei* (PGT13) and *T. harzianum* (PGT4)) and co-culture sample PGTA (*T. harzianum*, *T. reesei* and *T. reesei*) were inoculated into 250 mL Erlenmayer flasks containing 100 mL of potato dextrose broth medium (PDB, HIMEDIA) supplemented with chloramphenicol antibiotics, incubated in static condition for 9 days at 25 °C [42]. After the incubation period, to avoid fragmentation of the mycelium, it was removed using a microbial loop and the cultures were filtered under vacuum through filter paper (Whatman No. 4). The final filtrate was called a crude extract of secondary metabolites. A control assay was performed to ensure an optimum filtration (to check for the absence of conidia and mycelia) procedure by spreading 30 µL of the final filtrate on petri plates under sterile conditions containing PDA medium. The plates were examined for fungal growth [41].

### 2.8. Extraction and Identification of Secondary Metabolites

The secondary metabolites were extracted by a solvent extraction method from filtrates, where ethyl acetate and filtrate in a ratio of 1:1 (*v*/*v*) was used to extract exhaustively. The upper layer of the solvent contains the compounds which were collected separately from the aqueous layer (PDB medium) using a separation funnel. The solvent ethyl acetate was evaporated from the filtrate using a vacuum rotary evaporator at 40 °C, 70 rpm until the extract got reduced to 4 mL; it was maintained at −20 °C in the deep freezer until further use. Ethyl acetate extracts were analyzed by Gas Chromatography Mass Spectroscopy (GC-MS) analysis. The GC-MS analysis was performed using Thermo Scientific, Ceres 800, MS DSQ II (Waltham, MA, USA) and the silica column was packed with Elite-5MS (5% biphenyl 95% dimethylpolysiloxane, 30 m × 0.25 mm ID × 250 μm df). For the separation of components, helium gas was used as a carrier gas, which maintained the constant flow of 1 mL/min. The temperature of the injector was maintained at 260 °C, which was set for the chromatographic run. The sample of 1 μL of extract was injected into the instrument where the temperature of the oven was 60 °C (2 min), followed by 300 °C at the rate of 10 °C·min^−1^ and 300 °C for 6 min. The conditions for the mass detector were a temperature of 230 °C for the transfer line, a temperature of 230 °C for the ion source, an ionization mode electron impact at 70 eV, a scan time of 0.2 s and a scan interval of 0.1 s. The comparison of spectrum of components were carried out with the database of spectrum of known components stored in the GC-MS NIST (2008) library [43].

### 2.9. In Vitro Screening of Secondary Metabolites Produced from Mono and Co-Culture of Trichoderma spp. for Its Antibacterial Activity by Agar Well Diffusion Method against Xanthomonas oryzae pv. oryzae (Xoo)

The *Xanthomonas oryzae* pv. *oryzae* (Xoo) bacterial cultures were inoculated to 100 mL conical flasks containing nutrient broth and were incubated at 28 °C overnight. The petri plates were swabbed with Xoo cultures whose concentration was adjusted to 10^8^ CFU/mL. The agar petri plates were made with the required number of wells using a sterile cork borer, ensuring the proper distribution of wells in the periphery and one in the center. Tetracycline was used as a positive control and distilled water as a negative control. The secondary metabolite crude extracts were loaded in each well. The plates were incubated at 28 °C for 24 h and after incubation, the plates were observed, and the results were recorded [44].

### 2.10. Mycoynthesis of Zinc Oxide Nanoparticles (ZnO NPs)

The green synthesis method was used to prepare ZnO NPs using the following fungi: *T. harzianum, T. reesei, T. reesei* and a co-culture of *Trichoderma* spp. The zinc nitrate hexahydrate (Zn(NO_3_)_2_·6H_2_O) was utilized within the test without any further purification, which was procured from Sigma-Aldrich analytical grade. The Zn(NO_3_)_2_·6H_2_O taken in 1 g were dissolved in 10 mL of double-distilled water to the above mixture of 2 mL of fungal extract *T. harzianum, T. reesei, T. reesei* and co-culture of *Trichoderma* spp. was added and stirred for ~5−10 min using a magnetic stirrer. The mixture obtained was kept in a preheated muffle furnace, which was maintained at 400 ± 10 °C at which temperature the reaction mixture boils, froths, and heat-forming foam dehydrates in less than 3 min. The product obtained was calcinated at 700 °C for 2 h, and the obtained final product was used for further studies [30,45].

### 2.11. Characterization of Mycosynthesized Zinc Oxide Nanoparticles

The UV-visible spectrophotometer (SL 159 ELICO) was used for recording the UV-visible absorption in the samples. The particles were then characterized by evaluating their chemical composition through FTIR spectroscopy. Powder X-ray diffractometer (Shimadzu) using Cu Kα (1.5418 Å) radiation with a nickel filter was used to examine the phase purity and the crystallinity of the superstructures. The surface morphology of the nanoparticles was examined by SEM (Hitachi Tabletop TM-3000) along with EDAX. Studies were carried out using JEOL JEM 2100 TEM transmission electron microscopy (TEM), high-resolution transmission electron microscopy (HRTEM), and selected area electron diffraction (SAED) [46].

### 2.12. Antibacterial Activity of Mycosynthesized ZnO NPs against Xanthomonas oryzae

A disc diffusion method was carried out to assess the presence of antibacterial activity of ZnO NPs. A bacterial culture of 0.5 McFarland standard was used to lawn Muller Hinton agar plates evenly using a sterile swab. The discs loaded with ZnO NPs 1 mg/mL (PGT4, PGT5, PGT13 and PGTA) were placed on the Mueller Hinton agar plates. Each test plate had a positive control, a negative control and four treated ZnO NP (PGT4, PGT5, PGT13 and PGTA) discs. The standard tetracycline (0.5 mg/mL) was used as a positive control, and the negative control used was distilled water. The plates were incubated at 28 ± 2 °C for 24 h. After the incubation, the plates were examined and results were recorded in mm [47].

The synthesized ZnO NPs were subjected for antibacterial activity by minimum inhibitory concentration (MIC), which was determined using a broth microdilution method with minor modifications [30]. The desired different concentrations in 100 µg/mL, 50 µg/mL, 25 µg/mL, 12.5 µg/mL and 6.25 µg/mL was obtained by diluting the ZnO NP stock solution (1 mg/mL) with dilution in Mueller Hinton Broth (MHB) medium, which was added to a 96-well sterile microtiter plate. The bacterial suspension measuring 10 µL was added to each well, which were then incubated for 24 h at 28 °C. MHB served as a negative control, the positive control being the tetracycline at the concentration of 100 μg/mL; all these tests were conducted in triplicates. After 24 h of incubation, the addition of 20 μL of iodonitrotetrazolium chloride dye (INT) (0.5 μg/mL) to each well determined the MIC values of the synthesized ZnO NPs. The microtiter plates were incubated for 60 min at 28 °C. MICs determine the lowest concentration of the drug that prevents the change of color from being colorless to red, where colorless tetrazolium salt acts as an electron acceptor and gets reduced to a red-colored formazan product by biologically active organisms [30].

### 2.13. Statistical Analysis

All data of antibacterial experiments were analyzed statistically, using SPSS software (version 20.0) and Microsoft Excel. The obtained data were further subjected to analysis of variance (ANOVA), and the means were analyzed using Duncan’s new multiple range post test at *p* ≤ 0.05.

## 3. Results and Discussion

### 3.1. Collection and Isolation of Bacteria from Diseased Rice Leaf

The infected leaf samples were collected from different districts of Karnataka like Kolar, Chikkaballapura, Mysuru, Tumkur, Mandya and Bellary; thirty-five bacterial isolates were isolated from samples of 85 different infected samples collected from different rice fields of Karnataka (Table 1). The Xoo was recovered from the samples collected, showing typical Xoo bacterial colony characteristics such as a yellow color and mucoid, convex colonies on plating the samples as explained by Jabeen et al. [33] (Table S1)

### 3.2. Identification of Isolated Bacteria by Biochemical and Molecular Characterization

Morphologically identified samples were pure cultured and further used for biochemical and physiological identification methods according to [33] for the tests such as Gram’s reaction, oxidase test, catalase test, KOH test, starch hydrolysis test, casein hydrolysis test, gelatin liquefaction test and pectin hydrolysis test. The isolated bacteria were Gram-negative, short rods producing yellow-colored pigment. The bacterial isolates tested were positive for catalase, oxidase, 3% KOH, gelatin liquefaction, starch hydrolysis and pectin hydrolysis. Our results correlate with the results of Arshad et al. [48] The isolates were positive for all the tests except for the Gram’s reaction, which showed it to be negative, indicating that it is a gram-negative organism. The identification of the isolated bacteria was confirmed to be *Xanthomonas oryzae pv. oryzae* (Xoo) by molecular analysis. The isolates were sub cultured and maintained for further antibacterial studies.

### 3.3. Collection of Rhizospheric Soil Samples

A total of 180 rhizospheric soil samples were collected from different crop-growing regions of Karnataka districts like Chikkaballapura, Kolar, Ramanagara and Tumkur and are shown in Table S2.

### 3.4. Isolation and Identification of Selected Trichoderma spp. by Molecular Characterization

The colonies of *Trichoderma* spp. were grown on PDA plates by incubating for 6–7 days at 28 °C. A total of 35 isolates of *Trichoderma* spp. were isolated from 197 different rhizosphere soils collected from different parts of Karnataka. Standard techniques were used for DNA isolation, agarose gel electrophoresis and PCR amplification. Three fungal cultures of *Trichoderma* spp. were identified by molecular identification and were found to be *Trichoderma harzianum* (PGT4) isolated from *Cucumis sativus* (cucumber) rhizosphere soil, *Trichoderma reesei* (PGT5) isolated from *Solanum melongena* (brinjal) rhizosphere soil and one more strain of *Trichoderma reesei* (PGT13) isolated from *Coriandrum sativum* (coriander) rhizosphere soil sample collected from the Chikkaballapura district. These isolates were further confirmed by database searches which were carried out with the BLAST programs available at the National Centre for Biotechnology Information (Bethesda, MD, USA), which showed the identification matching to 99%, 100% and 100%, respectively. Our findings co-related with that of Ru and Di [37], where the isolates were isolated from rhizosphere soil of potato (Table S3).

### 3.5. In Vitro Screening of PGP Strains of Trichoderma spp. for Its Antibacterial Activity by Agar Plug Method against Xanthomonas oryzae pv. oryzae (Xoo)

The control of a broad range of plant pathogens, including fungal, bacterial and viral diseases, through elicitation of Induced Systemic Resistance (ISR) by *Trichoderma* spp. or localized resistance has been reported. Some *Trichoderma* spp. rhizosphere-competent strains have been shown to have direct effects on plants, increasing their growth potential and nutrient uptake, fertilizer use efficiency, percentage and rate of seed germination and stimulation of plant defenses against biotic and abiotic damage by Hermosa et al. [49]. A total of 35 *Trichoderma* spp. were subjected for preliminary antibacterial activity screening on solid media. The plates were pre swabbed with Xoo and the agar plugs of 7 mm of fungi taken from an actively growing region of 7-day-old culture were placed on the Mueller Hinton agar (MHA). The plates were kept for incubation in the incubator for 24 h at 28 °C. After the incubation period, the plates were observed for zone of inhibition and in each plate, a standard tetracycline was used as a positive control and distilled water was taken as a negative control. All the isolates showed activity against the tested three plant pathogenic bacteria Xoo. The majority of these isolates showed a wide range of inhibition of the Xoo cultures. The zone of inhibition ranged from 11 mm to 25 mm. There are reports of *Trichoderma harzianum* isolate which showed strong antagonism against fungal species by Leelavathi et al. [50] (Figure S1) (Table 1). Based on the zone of inhibition, three *Trichoderma* spp. were selected for further analysis.

### 3.6. Trichoderma–Trichoderma Interactions through Co-Culture

**Figure 1:**
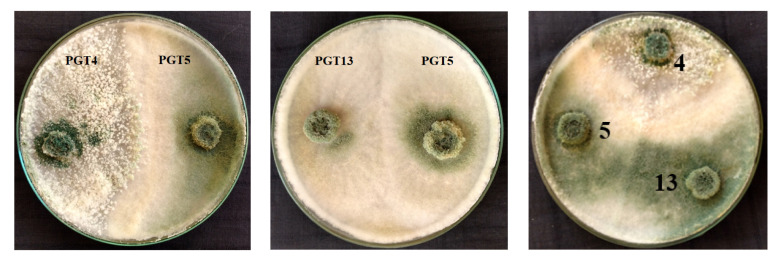
Co-culture of *Trichoderma* spp. plates showing the compatibility: 4—*Trichoderma harzianum*, 5—*Trichoderma reesei* and 13—*Trichoderma reesei*.

The co-culture of selected *Trichoderma* spp. isolates showed the production of secondary metabolites on incubation, showing growth compatibility when grown on solid media. However, no dual cultures were found to be incompatible. Our findings co-related with the results of that of the development of secondary metabolites on a laboratory scale; fungal isolates containing the largest areas of secondary metabolite accumulation on mono and dual cultures on semi-solid media were used by Ortuno et al. [41] (Figure 1).

### 3.7. Identification of Secondary Metabolite Compounds by GC-MS Analysis

**Figure 2:**
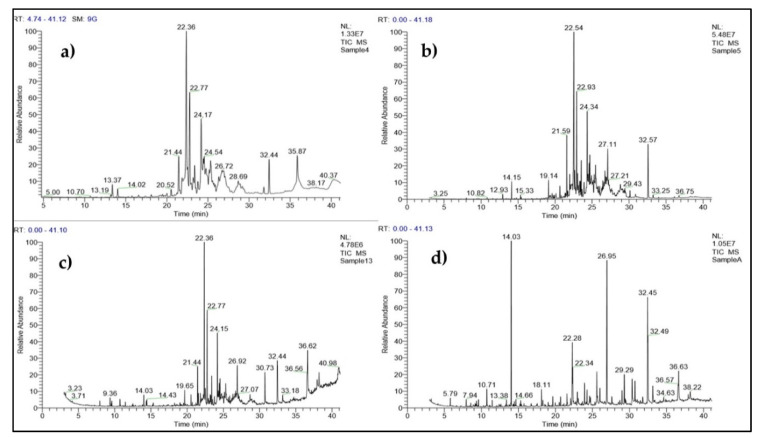
(**a**–**d**) Gas Chromatography Mass Spectroscopy (GC-MS) of compounds identified from secondary metabolite crude extracts of (**a**) *T. harzianum* (PGT4), (**b**) *T. reesei* (PGT5), (**c**) *T. reesei* (PGT13) and (**d**) co-culture of *Trichoderma* spp. (PGTA).

The ethyl acetate extract of the monocultures of *T. harzianum* (PGT4), *T. reesei* (PGT5) and *T. reesei* (PGT13) and co-cultures of Sample A (PGT4, PGT5, PGT13) were analyzed by GC-MS and the analysis has led to the identification of different compounds present in mono and co-culture of *Trichoderma* spp. Co-cultivation of beneficial fungi can stimulate the synthesis of novel secondary metabolites rather than in monocultures. The significant compounds that were found in the ethyl acetate solvent extract for PGT4 (Table 2), PGT5 (Table 3) and PGT13 (Table 4) and Sample A (Table 5). Gas chromatographic (GC) methods are usually done for the determination of volatile fungal metabolites for different fungi such as *Aspergillus, Fusarium, Mucor, Penicillium* and *Trichoderma* (Siddiquee et al. [51] (Figure 2)).

Interpretation of Mass Spectrum Gas Chromatography Mass Spectroscopy (GC-MS) was carried out using the database of the National Institute of Standard and Technology (NIST) with more than 62,000 patterns. The unknown spectrum of components was compared with that of the known components contained in the NIST library. The name, molecular weight and structure of the components of the test material were ascertained.

### 3.8. In Vitro Screening of PGPR Trichoderma spp. Co-Culture Secondary Metabolites for Its Antibacterial Activity by Agar Well Diffusion Method against Xanthomonas oryzae *pv.* oryzae *(Xoo)*

In our study, we analyzed the effects of growing the fungal cultures in single cultures or in combination cultures of *T. harzianum* (PGT4), *T. reesei* (PGT5) and one more culture of *T. reesei* (PGT13) for the production of fungal secondary metabolites in liquid media.

**Figure 3:**
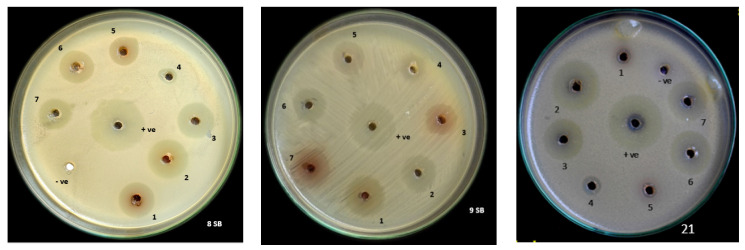
Screening of *Trichoderma* spp. co-culture secondary metabolites for its antibacterial activity against plant pathogen *Xanthomonas oryzae* pv. *oryzae* (Xoo); 8SB, 9SB and 21 represent strains MBXoo69, MBXoo70 and MBXoo53, respectively.

The zone of inhibition was found to range from 26 mm to 29 mm in diameter for the co-culture of (PGTA) followed by *T. harzianum* (PGT4) and *T*. *reesei* (PGT13) ranging from 23 mm to 26 mm in diameter, followed by a mono culture of *T. reesei* (PGT13). The zone of inhibition for *T. reesei* (PGT5) and *T. reesei* (PGT13) ranged from 20 mm to 26 mm. The zone of inhibition was found to be highest in the co-culture rather than in the mono cultures. Our findings co-related with the results of *T. harzianum* M10 and *T. pinophilus* F36CF on the production of fungal secondary metabolites in the liquid culture both in single and combined treatment by Vinale et al. [22] (Figure 3) (Table 6).

### 3.9. Characterization of Synthesized Nanoparticles

**Figure 4:**
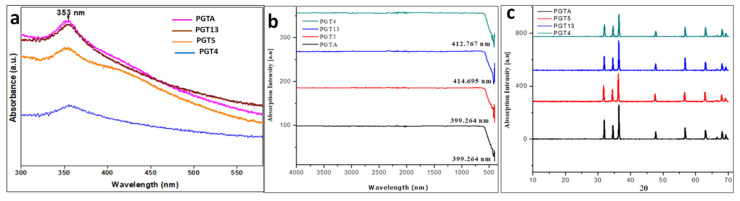
(**a**). UV−vis spectra of co-culture of *Trichoderma* sp. (PGTA), *Trichoderma reesei* (PGT5), *Trichoderma reesei* (PGT13) and *Trichoderma harzianum* (PGT4); (**b**) FT-IR Spectrogram of synthesized ZnO NPs *Trichoderma harzianum* (PGT4), *Trichoderma reesei* (PGT13), *Trichoderma reesei* (PGT5) and co-culture of *Trichoderma* sp. (PGTA); (**c**) PXRD patterns of ZnO NPs of *Trichoderma* spp.; co-culture of *Trichoderma* spp. (PGTA), *Trichoderma reesei* (PGT5), *Trichoderma reesei* (PGT13) and *Trichoderma harzianum* (PGT4).

The UV-visible absorption spectra was observed within the range of 372–374 nm. The obtained results matched with that of the UV–vis spectra of ZnO NPs for olive leaves (*Olea europaea*), chamomile flower (*Matricaria chamomilla* L.) and red tomato fruit (*Lycopersicon esculentum* M.) and showed strong absorption bands at 384, 380 and 386 nm respectively according to Ogunyemi et al. [52] (Figure 4a).

The FTIR spectrum showed the absorption at 400 cm ^−1^ to 600 cm ^−1^ which further confirms the presence and formation of ZnO nanoparticles by using *Trichoderma* spp. Similar results were observed from nanoparticles synthesized by the biological method using plant extracts Ogunyemi et al. [52] At 700 °C, the obtained fungal secondary metabolites were converted to their respective oxides, leading to the formation of ZnO NPs. This implies that most of the compounds present in the sample do not have a high thermal stability. Hence there were no other vibration modes detected in the FT-IR spectra as shown in (Figure 4b) other than ZnO NPs.

The biosynthesized ZnO NPs PXRD patterns showed noticeable peaks and it was well-matched to JCPDS No. 75-576. Similarly, nanoparticles from *Trichoderma* spp. are synthesized with ZnO. The biosynthesized ZnO-NPs of the crystalline structure was confirmed by stiff and narrow diffraction peaks with no significant variance in the diffraction peaks, suggesting that the crystalline product was free of impurities. Similarly, Lakshmeesha et al. [30] reported the green synthesis of *Nerium oleander* ZnO-NPs with no impurities in the obtained crystalline product. The size of the present study’s crystalline particles of green synthesized ZnO-NPs was calculated using Scherrer’s formula, which was within a range of 12–35 nm. Accordingly, Dobrucka and Dlugaszewska [53] reported the biosynthesis of ZnO nanoparticles using *Trifolium pratense*, with a hexagonal wurtzite shape and the sharp peaks calculated using Scherrer’s formula were 60–70 nm according to Murali et al. [54] (Figure 4c).

**Figure 5:**
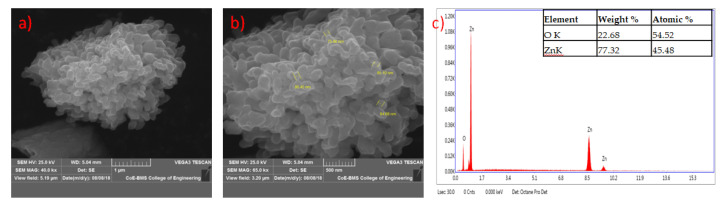
SEM image of zinc oxide nanoparticles synthesized from *Trichoderma harzianum* (PGT4) at lower (**a**) and higher magnification (**b**); (**c**) represents energy-dispersive X-ray spectroscopy (EDAX) analysis.

**Figure 6:**
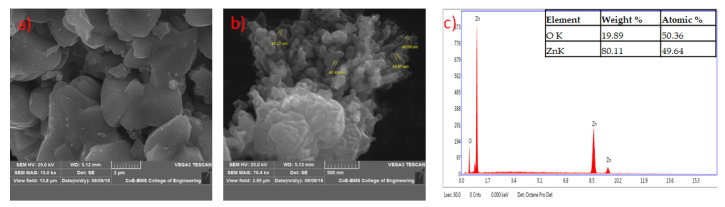
SEM image of zinc oxide nanoparticles synthesized from *Trichoderma reesei* (PGT5) at lower (**a**) and higher (**b**) magnification; (**c**) represents energy-dispersive X-ray spectroscopy (EDAX) analysis.

**Figure 7:**
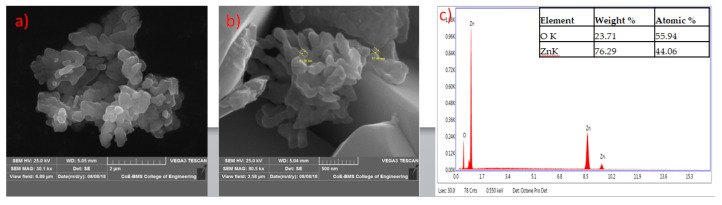
SEM image of zinc oxide nanoparticles synthesized from *Trichoderma reesei* (PGT13) at lower (**a**) and higher (**b**) magnification; (**c**) represents energy-dispersive X-ray spectroscopy (EDAX) analysis.

**Figure 8:**
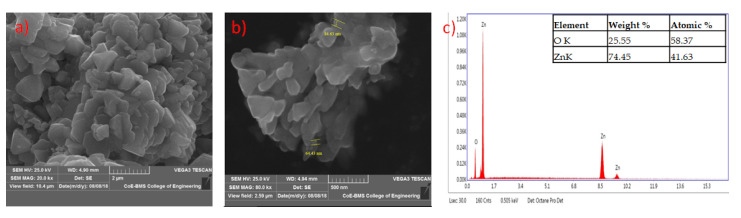
SEM image of zinc oxide nanoparticles synthesized from *Trichoderma* spp. co-culture of (PGTA) at lower (**a**) and higher (**b**) magnification; (**c**) represents energy-dispersive X-ray spectroscopy (EDAX) analysis.

The formed shapes of ZnO NPs were displayed in SEM images with different surface morphology. The elements involved in the formation of nanoparticles were subjected for the EDAX analysis to know the qualitative difference as well as the quantitative difference. The analysis revealed the highest proportion of zinc (50.36%) in nanoparticles and oxygen (49.64%) in all the synthesized nanoparticles according to Prasad et al. [55] (Figure 5, Figure 6, Figure 7 and Figure 8).

**Figure 9:**
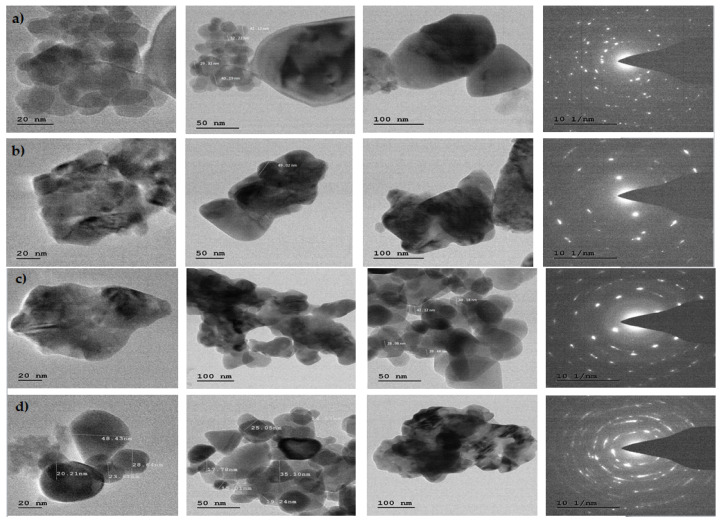
TEM micrographs (at different magnifications) and SAED patterns of Zno NPs synthesized from (**a**) *Trichoderma harzianum* (PGT4); (**b**) *Trichoderma reesei* (PGT5); (**c**) *Trichoderma reesei* (PGT13); and (**d**) *Trichoderma* spp. co-culture (PGTA).

TEM and SAED patterns correspond to ZnO compounds were obtained. The TEM image shows the agglomerated small particles of ZnO. The high resolution TEM image shows the well-defined crystal planes. The SAED patterns are well matched with the (hkl) values corresponding to the prominent peaks of the PXRD profiles (Figure 9a–d).

### 3.10. Antibacterial Activity

The antibacterial activity of ZnO NPs has been evaluated by measuring the zone of inhibition around the disc. The antibacterial activity of biosynthesized ZnO NPs was tested by an agar disc diffusion method, which was placed on the pre-swabbed Mueller-Hinton agar plate. The zone of inhibition is represented in Figure S2 and tabulated in Table 7. Further MIC values were determined for the biosynthesized ZnO NPs by the 96 well plate method, which is tabulated in Table 7 and is represented in Figure S3. The pronounced antibacterial activity of ZnO NPs can be due to its relatively small size and high surface-to-volume ratio. The present study clearly signifies the potentiality of ZnO NPs as antibacterial agents against Xoo (Figure S3) (Table 7). Our results correlated with the results of Ogunyemi et al. [52], where the zone of inhibition was recorded and the antibacterial activity of ZnO NPs was checked against Xoo when used in different concentrations.

## 4. Conclusions

Biological control provides an alternative for chemical fertilizers and reduces costs as well as environmental pollution. *Trichoderma* spp. are a good source of secondary metabolites. Co-cultivation of these beneficial fungi can stimulate the synthesis of novel secondary metabolites better than in monocultures. These *Trichoderma* cultures in combination can be used in field trials as they are able to inhibit the growth of the *Xanthomonas oryzae* pv. *oryzae* in in vitro conditions. The maximum zone of inhibition was recorded from the co-cultures rather than the monocultures. Biosynthesized ZnO NPs were also able to inhibit the growth of the *Xanthomonas oryzae* pv. *oryzae* in in vitro conditions, which was found to be dose-dependent.

## Figures and Tables

**Table 1:** Antibacterial activity of *Trichoderma* spp. isolated from rhizosphere soil on screening against *Xanthomonas oryzae* pv. *oryzae* (Xoo) (Mean ± standard deviation).

*Trichoderma* spp. Culture	Xoo Isolates (Zone of Inhibition)		
	MBXoo69 (mm in Diameter)	MBXoo70 (mm in Diameter)	MBXoo53 (mm in Diameter)
PGT1	20.33 ^mno^ ± 0.577	22.33 ^qrs^ ± 0.577	19.33 ^lmn^ ± 1.155
PGT2	19.67 ^lmn^ ± 0.577	21.00 ^mno^ ± 1.732	19.33 ^mno^ ± 1.155
PGT3	20.00 ^mnop^ ± 0.000	20.33 ^lmn^ ± 1.528	19.00 ^lmn^ ± 1.000
PGT4	18.33 ^jk^ ± 0.577	22.67 ^pqr^ ± 1.155	24.67 ^v^ ± 0.577
PGT5	19.67 ^lmn^ ± 0.577	22.00 ^pq^ ± 1.000	19.67 ^lmn^ ± 0.577
PGT6	19.33 ^lmn^ ± 0.577	21.33 ^pq^ ± 0.577	21.33 ^pqr^ ± 0.577
PGT7	19.67 ^lmn^ ± 0.577	19. 67 ^lmn^ ± 0.577	20.33 ^mno^ ± 0.577
PGT8	19.67 ^lmn^ ± 0.577	19.67 ^lmn^ ± 0.577	19.67 ^lmn^ ± 0.577
PGT9	19.67 ^lmn^ ± 0.577	19.67 ^lmn^ ± 0.577	20.00 ^mno^ ± 0.000
PGT10	19.67 ^lmn^ ± 0.577	17.67 ^ijk^ ± 0.577	19.67 ^lmn^ ± 0.577
PGT11	19.67 ^lmn^ ± 0.577	21.33 ^pq^ ± 0.577	19.67 ^lmn^ ± 0.577
PGT12	19.67 ^lmn^ ± 0.577	16.67 ^gh^ ± 0.577	20.00 ^mno^ ± 0.000
PGT13	20.00 ^mno^ ± 0.000	21.67 ^pqr^ ± 0.577	19.67 ^lmn^ ± 0.577
PGT14	19.67 ^lmn^ ± 0.577	21.67 ^pqr^ ± 0.577	19.67 ^lmn^ ± 0.577
PGT15	16.00 ^fg^ ± 0.000	18.33 ^jk^ ± 0.577	20.00 ^mno^ ± 0.000
PGT16	17.67 ^ijk^ ± 0.577	18.67 ^kl^ ± 0.577	19.67 ^lmn^ ± 0.577
PGT17	19.67 ^lmn^ ± 0.577	17.00 ^hi^ ± 0.000	19.33 ^lm^ ± 0.577
PGT18	17.67 ^ijk^ ± 0.577	14.67 ^cd^ ± 0.577	18.33 ^jk^ ± 0.577
PGT19	19.67 ^lmn^ ± 0.577	18.33 ^jk^ ± 0.577	18.00 ^jk^ ± 0.000
PGT20	19.67 ^lmn^ ± o.577	18.33 ^jk^ ± 0.577	19.67 ^lmn^ ± 0.577
PGT21	17.33 ^hij^ ± 0.577	18.33 ^jk^ ± 0.577	18.33 ^jk^ ± 0.577
PGT22	19.67 ^lmno^ ± 0.577	21.67 ^pqr^ ± 0.577	18.33 ^jk^ ± 0.577
PGT23	18.33 ^jk^ ± 0.577	19.67 ^lmno^ ± 0.577	19.67 ^lmno^ ± 0.577
PGT24	20.67 ^nop^ ± 0.577	19.67 ^lmn^ ± 0.577	20.00 ^mno^ ± 0.000
PGT25	14.00 ^c^ ± 0.000	21.67 ^pqr^ ± 0.577	14.67 ^cd^ ± 0.577
PGT26	12.33 ^b^ ± 0.577	18.33 ^jk^ ± 0.577	15.67 ^ef^ ± 0.577
PGT27	11.00 ^a^ ± 0.000	18.00 ^jk^ ± 0.000	15.00 ^de^ ± 0.000
PGT28	11.00 ^a^ ± 0.000	18.33 ^jk^ ± 0.577	15.00 ^de^ ± 0.000
PGT29	12.33 ^b^ ± 0.577	17.67 ^ijk^ ± 0.577	19.67 ^lmn^ ± 0.577
PGT30	24.67 ^v^ ± 0.577	24.67 ^v^ ± 0.577	20.67 ^nop^ ± 0.577
PGT31	22.67 ^st^ ± 0.577	23.67 ^u^ ± 0.577	21.00 ^opq^ ± 0.000
PGT32	24.67 ^v^ ± 0.577	25.00 ^v^ ± 0.000	21.67 ^pqr^ ± 0.577
PGT33	22.67 ^st^ ± 0.577	25.33 ^v^ ± 0.577	23.00 ^uv^ ± 0.000
PGT34	21.00 ^opq^ ± 0.000	21.67 ^pqr^ ± 0.577	19.67 ^lmno^ ± 0.577
PGT35	22.67 ^st^ ± 0.577	20.00 ^mn^ ± 0.000	20.67 ^nop^ ± 0.000
Positive	25.00 ^v^ ± 0.577	26.00 ^v^ ± 0.000	25.00 ^rs^ ± 0.000
Negative	0 ^a^	0 ^a^	0 ^a^

The mean values are recorded and the values are not significantly different according to Duncan’s Multiple Range Test indicated by different alphabets in the superscripts at *p* < 0.05.

**Table 2:** List of compounds of *Trichoderma harzianum* (PGT4) detected by Gas Chromatography Mass Spectroscopy (GC-MS). RT—Retention Time.

SL. No.	RT (mins)	Name of the Compound *Trichoderma harzianum* (PGT4)	Molecular Formula	Molecular Weight
1	10.70	4-Propylbenzaldehyde	C_10_H_12_O	148
2	13.37	6-Pentyl-2H-pyran-2-one	C_10_H_14_O_2_	166
3	14.02	2,4-Di-tert-butylphenol	C_14_H_22_O	206
4	15.76	4,6-*O*-Furylidene-d-glucopyranose	C_11_H_14_O_7_	258
5	16.55	Trimethyl-3,4-undecadiene-2,10-dione	C_14_H_22_O_2_	222
6	17.37	1,5-Diphenyl-3-(3-cyclopentylpropyl)pentane	C_25_H_34_	334
7	18.10	1-[2-Methyl-2-(-4-methyl-3-pentenyl)cyclopropyl]ethanol	C_12_H_22_O	182
8	21.44	Phthalic acid, diisobutyl ester	C_16_H_22_O_4_	278
9	22.36	Dibutyl phthalate	C_16_H_22_O_4_	278
10	22.77	Phthalic acid, butyl 2-pentyl ester	C_17_H_24_O_4_	292
11	23.38	Phthalic acid, 6-ethyl-3-octyl butyl ester	C_22_H_34_O_4_	362
12	24.17	Dibutyl phthalate	C_16_H_22_O_4_	278
13	31.79	3-Ethyl-3-hydroxyandrostan-17-one	C_21_H_34_O_2_	318
14	32.44	Mono(2-ethylhexyl) phthalate	C_16_H_22_O_4_	278
15	35.87	Digitoxin	C_41_H_64_O_13_	764

**Table 3:** List of compounds of *Trichoderma reesei* (PGT5) detected by GC-MS. RT—Retention Time.

SL. No.	RT (mins)	Name of the Compound *Trichoderma reesei* (PGT5)	Molecular Formula	Molecular Weight
1	8.02	n-Nonaldehyde	C_9_H_18_O	142
2	10.82	p-propylbenzaldehyde	C_10_H_12_O	148
3	12.95	4-(2-Hydroxyethyl)phenol	C_8_H_10_O_2_	138
4	14.13	Phenol, 2,4-di-tert-butyl	C_14_H_22_O	206
5	19.14	(3E)-3-Octadecene	C_18_H_36_	252
6	20.65	Phthalic acid, butyl isobutyl ester	C_16_H_22_O_4_	278
7	22.52	Dibutyl phthalate	C_16_H_22_O_4_	278
8	22.93	Phthalic acid, butyl 2-pentyl ester	C_17_H_24_O_4_	292
9	23.54	1,2-Benzenecarboxylic acid, bis(2-methylpropyl) ester	C_16_H_22_O_4_	278
10	24.34	Dibutyl phthalate	C_16_H_22_O_4_	278
11	27.11	1-Hydroxy-4-methylanthra-9,10-quinone	C_15_H_10_O_3_	238
12	28.82	3-Chloro-6-(phenylsulfsnyl)bicycle(3.1.1)hept-2-ene	C_13_H_13_CIS	236
13	32.57	Mono(2-ethylhexyl) phthalate	C_16_H_22_O_4_	278
14	33.27	(10E)-10-Henicosene	C_21_H_42_	294
15	21.59	Phthalic acid, diisobutyl ester	C_16_H_22_O_4_	278

**Table 4:** List of compounds of *Trichoderma reesei* (PGT13) detected by GC-MS. RT—Retention Time.

SL. No.	RT (mins)	Name of the Compound *Trichoderma reesei* (PGT13)	Molecular Formula	Molecular Weight
1	7.93	5,6-Dimethylundecane	C_13_H_28_	184
2	9.36	1-Methylene-1H-indene	C_10_H_8_	128
3	10.72	Benzaldehyde, 4-propyl	C_10_H_12_O	148
4	14.03	Phenol, 2,4-di-tert-butyl	C_14_H_22_O	206
5	14.35	Maleic acid, dibutyl ester	C_12_H_20_O_4_	228
6	15.31	Phthalic acid, ethyl 2-methylbutyl ester	C_15_H_20_O_4_	264
7	19.65	Tetradeconic acid, 1-methylethyl ester	C_17_H_32_O_2_	270
8	21.44	Phthalic acid, butyl isobutyl ester	C_16_H_22_O_4_	278
9	22.34	Dibutyl phthalate	C_16_H_22_O_4_	278
10	22.77	Phthalic acid, butyl 2-pentyl ester	C_17_H_24_O_4_	292
11	24.15	Phthalic acid, butyl hexyl ester	C_18_H_6_O_4_	306
12	26.92	Anthraquinone, 1-hydroxy-2-methyl	C_15_H_10_O_3_	238
13	30.73	Cis-4,7,10,13,16,19-Docosahexanoic acid, tert-butyldimethyldilyl ester	C_28_H_46_O_2_Si	442
14	32.46	9-t-Butyltricyclo[4.2.1.1(2,5)]decane-9-10-diol	C_14_H_24_O_2_	224
15	36.62	(5E,7E)-25-[(Trimethylsilyl)oxy]-9, 10-secocholesta-5,7,10-triene-1,3-diol	C_30_H_52_O_3_Si	488
16	38.21	2,6-Ditert-butyl-4-methylphenyl 2-methylcyclopropanecarboxylate	C_20_H_30_O_2_	302

**Table 5:** List of compounds of *Trichoderma* spp. co-culture (PGTA) detected by GC-MS. RT—Retention Time.

SL. No.	RT (mins)	Name of the Compound Sample A (4,5,13)	Molecular Formula	Molecular Weight
1	10.71	Benzaldehyde, 4-propyl	C_10_H_12_O	148
2	18.11	1-[2-Methyl-2-(-4-methyl-3-pentenyl)cyclopropyl]ethanol	C_12_H_22_O	182
3	5.79	4-Hydroxybenzenephophoric acid	C_6_H_7_O_4_P	174
4	7.94	Nonanal	C_9_H_18_O	142
5	14.03	Phenol, 2,4,-di-tert-butyl	C_14_H_22_O	206
6	21.55	1-Oxa-spiro[4,5]deca-6,9-diene-2,8-dione, 7,9-di-tert-butyl	C_17_H_24_O_3_	276
7	22.28	Hexadecanoic acid	C_16_H_32_O_2_	256
8	23.91	Chrysophanic acid anthranol	C_15_H_12_O_3_	240
9	25.58	Trans-13-Octadecenoic acid	C_18_H_34_O_2_	282
10	26.95	1-Hydroxy-4-methylanthra-9, 10-quinone	C_15_H_10_O_3_	238
11	29.29	10,12-Pentacosadiynoic acid	C_25_H_42_O_2_	374
12	30.35	4-(2-Oxiranyl)-9H-fluoren-9-ol	C_15_H_12_O_2_	224
13	32.47	9-t-Butyltricyclo[4.2.1.1(2,5)]decane-9,10-diol	C_14_H_24_O_2_	224
14	33.14	6,9-Octadecadiynoic acid, methyl ester	C_19_H_30_O_2_	290
15	36.61	Ledene oxide-(II)	C_15_H_24_O	220

**Table 6:** Antibacterial activity of *Trichoderma* spp. co-culture secondary metabolite against plant pathogen Xoo (Mean ± standard deviation).

SL. No.	*Trichoderma* spp. Coculture Secondary Metabolite	Xoo Isolates (Zone of Inhibition)		
		MBXoo69 (8SB) (mm in Diameter)	MBXoo70 (9SB) (mm in Diameter)	MBXoo53 (21) (mm in Diameter)
1	PGT13	24 ^defgh^ ± 1.00	25.33 ^gh^ ± 1.15	24.67 ^fgh^ ± 0.577
2	PGT4,5,13	22.33 ^cde^ ± 1.528	28 ^i^ ± 1.00	21.67 ^cd^ ± 1.528
3	PGT4,13	22 ^cd^ ± 2.000	24.67 ^gh^ ± 0.577	22.00 ^cd^ ± 1.000
4	PGT4	15.67 ^b^ ± 1.155	15.67 ^b^ ± 1.155	13.33 ^a^ ± 1.528
5	PGT5	20.33 ^c^ ± 0.577	23.33 ^def^ ± 1.155	24.33 ^efg^ ± 0.577
6	PGT5,13	20.67 ^c^ ± 0577	23.33 ^def^ ± 1.528	21.67 ^cd^ ± 1.528
7	PGT4,5	21.67 ^cd^ ± 0.577	21.67 ^cd^ ± 0.577	20.67 ^c^ ± 0.577
Positive	Positive	26.00 ^gh^ ± 1.732	29.33 ^hi^ ± 1.155	27.67 ^i^ ± 0.577
Negative	Negative	0 ^a^	0 ^a^	0 ^a^

The mean values are recorded and the values are not significantly different according to Duncan’s Multiple Range Test indicated by different alphabets in the superscripts at *p* < 0.05.

**Table 7:** Evaluation of the bactericidal activity of biosynthesized zinc oxide nanoparticles from different species of *Trichoderma* against different strains of *Xanthomonas oryzae* pv. *oryzae* (Xoo) (Concentration expressed in μg/mL).

ZnO NPs	Disc Diffusion Values (in mm)	MIC Values (µg/mL)
PGT 4	00	50
PGT5	14.33 ± 0.33	25
PGT13	00	50
PGTA	15.67 ± 0.33	25
Positive	13.67 ± 0.33	25
Negative	00	00

## Supplementary Materials

The following are available online at https://www.mdpi.com/2309-608X/6/3/181/s1, Figure S1. Screening of fungal discs (*Trichoderma* spp.) for antibacterial activity against *Xanthomonas oryzae* pv. *oryzae* A- MBXoo69, B- MBXoo53 (+- tetracycline - distilled water). Figure S2: Antibacterial activity of zinc oxide nanoparticles (ZnO NPs) against *Xanthomonas oryzae* pv. *oryzae*(Xoo) by disc diffusion method (+ ve = tetracycline; − ve = distilled water). Figure S3: Bacterial sensitivity test of biosynthesized zinc oxide nanoparticles from *Trichoderma harzianum* (PGT4) (MH429899.1), *Trichoderma reesei* (PGT5) (MH429901.1), *Trichoderma reesei* (PGT13) (MH429900.1) and co-culture of *Trichoderma* sp. (PGTA) against different strains of plant pathogen *Xanthomonas oryzae* pv. *oryzae* (MF579736.1) (concentration in μg/ml) (a- Trail 1, b- Trail 2, and c- Trail 3). Table S1: Place of collection of infected samples from different crop-growing regions of Karnataka. Table S2: Places of Rhizospheric soil sample collection. Table S3: Morphological and physiology characters of Rhizosphere Trichoderma spp. fungi.
