# Supplementary material for: Mycosynthesis of ZnO Nanoparticles Using Trichoderma spp. Isolated from Rhizosphere Soils and Its Synergistic Antibacterial Effect against Xanthomonas oryzae pv. oryzae
Source: J Fungi (Basel). 2020 Sep 20;6(3):181. doi: 10.3390/jof6030181 (PMC7558757; doi:10.3390/jof6030181)
Supplement: Supplementary file 1 [file jof-06-00181-s001.pdf]

Supplementary Materials

Table S1: Place of collection of infected samples from different crop-growing regions of Karnataka.

District and No. of isolates	Plot place No.	Rice cultivar
**Kolar (39)**	Diguvapalli	Hamsa
	Hosahalli	Kaveri
	Lakshmipura	Hamsa
	Nernahalli	Hamsa
	Palurahalli	Kaveri
	Gollahalli	Hamsa
**Chikkaballapura (24)**	Chintamani	Jaya
	Shidlaghatta	Kaveri
	Siddepalli	Hamsa
	Birur	Hamsa
	Gauribidnur	Jaya
**Mysore (10)**	Periyapatna	Rjabhoga
	Nanjangudu	Rajamudi
	Saligrama	MTU 1001
	Hunsur	Thanu
	Bannur	Jyothi
**Tumkur (8)**	Thuruvekere	Sona
	Kunigal	Hamsa
	Honnenahalli	Jaya

	Hokkodi	IR 20
	Bettadahalli	Sona
**Mandya (0)**	Pandavapura	Mandya Vijaya
	Mandagere	IR 20
	Haralahalli	BR
	Malavalli	Naga rice
	Shivalli	IR 20
**Bellary (4)**	Hospet	Sonamasuri
	Siruguppa	Sonamasuri

Table S2: Places of Rhizospheric soil sample collection

Place and No. of Rhizospheric soil samples collected	*Trichoderma* spp.	Plant
**Chikkaballapura**		
Adavigollavarahalli (2)	PGT1	1.*Eleusine coracana*(Ragi)<br>2.*Solanum lycopersicum(Tomato)*
Addagal (4)	PGT2	3.*Macrotyloma uniflorum*(Horse gram)<br>4.*Eleusine coracana*(Ragi)<br>5.*Lablab purpureus (*Hyacinath beans)<br>6.*Eleusine coracana*(Ragi)
Ittapanahalii (2)	PGT3	7.*Phaseolus vulgaris* (Beans)<br>8.*Zea mays*(Maize)
Hosahalli (3)		9.*Vigna unguiculata*(Cowpea)<br>10. *Cajanus Cajana (L) Millsp*.(Pigeon pea)<br>11. *Eleusine coracana*(Ragi)
Gollahalli (5)		12. *Mintha piperita*(Mint leaves)<br>13. *Macrotyloma uniflorum*(Horse gram)<br>14. *Vigna unguiculata(Cowpea)*<br>15. *Brassica nigra (*Mustard)<br>16. *Cajanus Cajana (L)Millsp*.(Pigeon pea)
Andrahalli (2)	PGT4	17. *Cucumis sativus* (Cucumber)<br>18. *Oriza sativa(*Rice)
Bommahalli (4)	PGT5	19. *Eleusinecoracana*(Ragi)<br>20. *Solanum melongena* (Brinjal)<br>21. *Phaseolus vulgaris* (Beans)<br>22. *Solanum melongena(*Brinjal
Arikere (6)	PGT6	23. *Brassica oleracea var. capitata*(Cabbage<br>24. *Vigna unguiculata*(Cowpea)<br>25. *Eleusinecoracana*(Ragi)

		26. *Punica granatum* (Pomegranate)<br>27. *Raphanus sativus*(Raddish)<br>28. *Solanum lycopersicum*(Tomato)
Bandahalli (4)		29. *Eleusine*coracana (Ragi)<br>30. *Raphanus sativus*(Raddish)<br>31. *Cajanus cajana (L)Millsp*.(Pigeon pea)<br>32. *Lablab purpureus (*Hyacinath beans)
Lakshmipathihalli (3)	PGT7	33. *Daucus carota* subsp. *sativus* (Carrot)<br>34. *Eleusinecoracana*(Ragi)<br>35. *Cajanus cajana (L) Millsp*.(Pigeon pea)
Chinthamani (5)	PGT8	36. *Eleusine coracana (*Ragi)<br>37. *Phaseolus vulgaris* (Beans)<br>38. *Lablab purpureus (*Hyacinath beans)<br>39. *Zea mays (*Maize)<br>40. *Solanum lycopersicum (*Tomato
Battalahalli (2)		41. *Solanum lycopersicum*(Tomato)<br>42. *Ricinus communis*(Castor)
**Mudbagal**		
Audugodi (4)	PGT9	43. *Phaseolus vulgaris* (Beans)<br>44. *Solanumlycopersicum(Tomato*)<br>45. *Zea mays* (Maize)<br>46. *Eleusinecoracana*(Ragi)
Nangali (4)	PGT10	47. *Benincasa hispida* (Ash guard)<br>48. Solanumlycopersicum (Tomato)<br>49. *.Daucuscarota subsp. sativus*(Carrot)<br>50. *Solanum lycopersicum* (Tomato)
Hanumanahalli (4)	PGT11	51. *Arachis hypogaea*(Groundnut)<br>52. *Momordica charantia*(Bitter gourd)<br>53. *Brassica oleracea* var. *botrytis* (Cauli flower)<br>54. *Solanum tuberosum*(Potato
Byrakur (3)	PGT12	55. *Cajanus Cajana (L) Millsp*.(Pigeon pea)<br>56. *Zea mays (*Maize)<br>57. *Zingiber officinale* (Ginger)
Mustur (3)		58. *Eleusine coracana*(Ragi)<br>59. *Coriandrum sativum*(Corriander leaves)<br>60. *Momordica charantia*(Bitter gourd)
Uttanur (5)	PGT13	61. *Allium cepa*(Onion)<br>62. *Coriandrum sativum* (Coriander)<br>63. *Allium sativum*(Garlic)<br>64. *Solanum melongena*(Brinjal)<br>65. *Eleusine coracana (*Ragi)
Hebbani (4)		66. *Eleusine coracana*(Ragi)<br>67. *Cajanus Cajana (L) Millsp*.(Pigeon pea)<br>68. *Eleusinecoracana* (Ragi)<br>69. Solanum *lycopersicum(Tomato*)
	**Ramanagara**	
Kailancha (3)		70. *Zingiber officinale*(Ginger)<br>71. *Luffa acutangula*(Ridge gourd)<br>72. *Lablab purpureus*(Hyacinth beans)
K P Doddi (3)	PGT14	73. *Solanum lycopersicum*(Tomato)<br>74. *.Eleusine coracana*(Ragi)<br>75. *Lablab purpureus (*Hyacinath beans)

Acchalu (2)	PGT15	76.	*Punica granatum* (Pomegranate)
		77.	*Zea mays* (Maize
Ramohalli (3)	PGT16	78.	*Vigna unguiculata* (Cowpea)
		79.	*Cajanus Cajana (L) Millsp.*(Pigeon pea)
		80.	*Eleusinecoracana* (Ragi)
Avarehalli (2)		81.	*Arachis hypogaea*(Groundnut)
		82.	*Momordica charantia*(Bitter gourd)
Gowdaiahna Doddi (3)		83.	*Cajanus Cajana (L) Millsp.*(Pigeon pea)
		84.	*Lablab purpureus (*Hyacinath beans)
		85.	*Solanum lycopersicum*(Tomato)
Kempegowda Doddi (3)	PGT17	86.	*Brassica* (Mustard)
		87.	*Solanum lycopersicum* (Tomato)
		88.	*Eleusine coracana*(Ragi)
Motte Doddi (3)	PGT18	89.	*Lablab purpureus (*Hyacinath beans)
		90.	*Momordica charantia* (Bitter guard)
		91.	*.Daucus carota subsp.*
Manchanmele (5)	PGT19	92.	*Solanum lycopersicum*(Tomato)
		93.	*Eleusine coracana*(Ragi)
		94.	*Zea mays*(Maize)
		95.	*Murraya koenigii* (Curry leaves)
		96.	*Lablab purpureus (*Hyacinath beans)
Kengal (2)	PGT20	97.	*Abelmoschus esculentus* (Lady's finger)
		98.	*Solanum tuberosum*(Potato)
Harohalli (5)	PGT21	99.	*Zea mays* (Maize)
		100.	*Lablab purpureus (*Hyacinath beans)
		101.	*Vigna unguiculata*(Cowpea)
		102.	*Eleusine coracana (Ragi)*
		103.	*Eleusine coracana*(Ragi)
Bheemasandhra (2)	PGT22	104.	*Arachis hypogea* (Groundnut)
		105.	*Solanum lycopersicum* (Tomato)
Girenahalli (2)		106.	*Eleusinecoracana*(Ragi)
		107.	*Arachis hypogaea*(Groundnut)
Thamsandra (1)		108.	*Macrotyloma uniflorum*(Horse gram)
Mudenahalli (3)	PGT23	109.	*Brassica* (Mustard)
		110.	*Phaseolus vulgaris* (Beans)
		111.	*Solanum melongena(*Brinjal)
Marasandhra (2)	PGT24	112.	*Phaseolus vulgaris* (Beans)
		113.	*Cajanus cajana (L) Millsp.*(Pigeon pea)
Kebbedhoddi (4)	PGT25	114.	*Zea mays* (Maize)
		115.	*Coriandrumsativum*(Corriander leaves)
		116.	*Cucurbita* (Pumpkin)
		117.	*Zea mays* (Maize)
DhoddaSadhanahalli (5)	PGT26	118.	*Raphanus sativus*(Raddish)
		119.	*Solanum lycopersicum*(Tomato)
		120.	*Allium cepa*(Onion)
		121.	*Arachis hypogaea*(Groundnut)
		122.	*Abelmoschus esculentus (Lady's finger)*
Gabbadi (2)		123.	*Solanumlycopersicum*(Tomato)
		124.	*Phaseolus vulgaris* (Beans)
**Tumkur**			
Baragur (3)		125.	*Zingiber officinale*(Ginger)
		126.	*Luffa acutangula*(Ridge gourd)
		127.	*Lablab purpureus*(Hyacinth beans)
Barakanallu (3)		128.	*Zingiber officinale*(Ginger)
		129.	*Luffa acutangula*(Ridge gourd)

		130.	*Lablab purpureus*(Hyacinth beans)
Bellagulla (3)	PGT27	131.	*Arachis hypogaea(Groundnut)*
		132.	*Ricinus communis (Castor)*
		133.	*Allium cepa*(Onion)
Chikanayakanhalli (4)	PGT28	134.	*Solanum tuberosum*(Potato)
		135.	*Eleusine coracana*(Ragi)
		136.	*Brassia (Mustard)*
		137.	*Beta vulgaris*(Beetroot)
Chowlakatte (5)	PGT29	138.	*Eleusine coracana*(Ragi)
		139.	*Phaseolus vulgaris* (Beans)
		140.	*Beta vulgaris*(Beetroot)
		141.	*Daucus carota subsp. sativus*(Carrot)
		142.	*Coriandrum sativum*(Corriander leaves)
Dasodi (3)		143.	*Zea mays* (Maize)
		144.	*Allium cepa*(Onion)
		145.	*Zingiber officinale*(Ginger)
Doddabidre (3)	PGT30	146.	*Cajanus Cajana (L) Millsp*.(Pigeon pea)
		147.	*Cucurbita* (Pumpkin)
		148.	Solanum *lycopersicum(Tomato*)
Doddayenegere (2)		149.	*Solanum lycopersicum*(Tomato)
		150.	*Ricinus communis*(Castor)
Dugudihalli (3)		151.	*Lablab purpureus (*Hyacinath beans)
		152.	*Arachis hypogaea*(Groundnut)
		153.	*Solanum tuberosum*(Potato)
Ganadallu (3)	PGT31	154.	*Solanum lycopersicum* (Tomato)
		155.	*Daucus carota subsp. sativus*(Carrot)
		156.	*Zea mays*(Maize)
		**Kolar**	
Bangarpet (3)		157.	*Cajanus Cajana (L) Millsp*.(Pigeon pea)
		158.	*Zea mays* (Maize)
		159.	*Solanum lycopersicum (*Tomato)
Mulbagal (3)	PGT32	160.	*Cajanus Cajana (L) Millsp*.(Pigeon pea)
		161.	*Zingiber officinale* (Ginger)
		162.	*Solanum lycopersicum (*Tomato)
Malur (1)		163.	*Allium sativum*(Garlic)
Srinivaspur (4)	PGT33	164.	*Vigna unguiculata*(Cowpea)
		165.	*Capsicum annum* (Green chilli)
		166.	*Solanum lycopersicum*(Tomato)
		167.	*Eleusine coracana*(Ragi)
Harohalli (3)		168.	*Eleusine coracana (*Ragi)
		169.	*Phaseolus vulgaris* (Beans)
		170.	*Oriza sativa* (Rice)
Bheemasandhra (4)	PGT34	171.	*Oriza sativa* (Rice)
		172.	*Momordica charantia* (Bitter guard)
		173.	*Eleusine coracana*(Ragi)
		174.	*Cajanus Cajana (L) Millsp*.(Pigeon pea)

Girenahalli (6)	PGT35	175.	*Benincasa hispida* (Ash guard)
		176.	*Cajanus Cajana (L) Millsp.*(Pigeon pea)
		177.	*Eleusinecoracana* (Ragi)
		178.	*Zea mays* (Maize)
		179.	*Solanum lycopersicum (*Tomato)
		180.	*Oriza sativa* (Rice)

Table S3: Morphological and physiology characters of Rhizosphere *Trichoderma* spp. fungi.

Place	Code	Plant	Colony colour
Mudbagal	PGT1	*Solanum lycopersicum* (Tomato)	Green
Ramanagara	PGT2	*Eleusine coracana* (Ragi)	Green
Chikkaballapura	PGT3	*Phaseolus vulgaris* (Beans)	Green
Mudbagal	PGT4	*Cucumis sativus* (Cucumber)	Green
Tumkur	PGT5	*Solanum melongena* (Brinjal)	Green
Ramanagara	PGT6	*Punica granatum* (Pomegranate)	Green
Tumkur	PGT7	*Daucus carota* subsp. *sativus* (Carrot)	Green
Chikkaballapura	PGT8	*Solanum lycopersicum* (Tomato)	Green
Chikkaballapura	PGT9	*Phaseolus vulgaris* (Beans)	Green
Mudbagal	PGT10	*Benincasa hispida* (Ash guard)	Green
Tumkur	PGT11	*Brassica oleracea* var. *botrytis* (Cauli flower)	Green
Chikkaballapura	PGT12	*Zingiber officinale* (Ginger)	Green
Chikkaballapura	PGT13	*Coriandrum sativum* (Coriander)	Green
Ramanagara	PGT14	*Eleusine coracana* (Ragi)	Green
Mudbagal	PGT15	*Zea mays* (Maize)	Green
Ramanagara	PGT16	*Vigna unguiculata* (Cowpea)	Green
Chikkaballapura	PGT17	*Brassica* (Mustard)	Green
Chikkaballapura	PGT18	*Momordica charantia* (Bitter guard)	Green
Ramanagara	PGT19	*Murraya koenigii* (Curry leaves)	Green
Mudbagal	PGT20	*Abelmoschus esculentus* (Lady's finger)	Green
Mudbagal	PGT21	*Zea mays* (Maize)	Green
Ramanagara	PGT22	*Solanum lycopersicum* (Tomato)	Green

Chikkaballapura	PGT23	*Brassica* (Mustard)	Green
Chikkaballapura	PGT24	*Phaseolus vulgaris* (Beans)	Green
Ramanagara	PGT25	*Cucurbita* (Pumpkin)	Green
Ramanagara	PGT26	*Abelmoschus esculentus* (Lady's finger)	Green
Chikkaballapura	PGT27	*Ricinus communis (Castor)*	Green
Mudbagal	PGT28	*Brassia (Mustard)*	Green
Mudbagal	PGT29	*Phaseolus vulgaris (Beans)*	Green
Ramanagara	PGT30	*Cucurbita (Pumpkin)*	Green
Chikkaballapura	PGT31	*Solanum lycopersicum (Tomato)*	Green
Chikkaballapura	PGT32	*Zingiber officinale* (Ginger)	Green
Ramanagara	PGT33	*Solanum lycopersicum* (Tomato)	Green
Ramanagara	PGT34	*Momordica charantia* (Bitter guard)	Green
Chikkaballapura	PGT35	*Benincasa hispida* (Ash guard)	Green

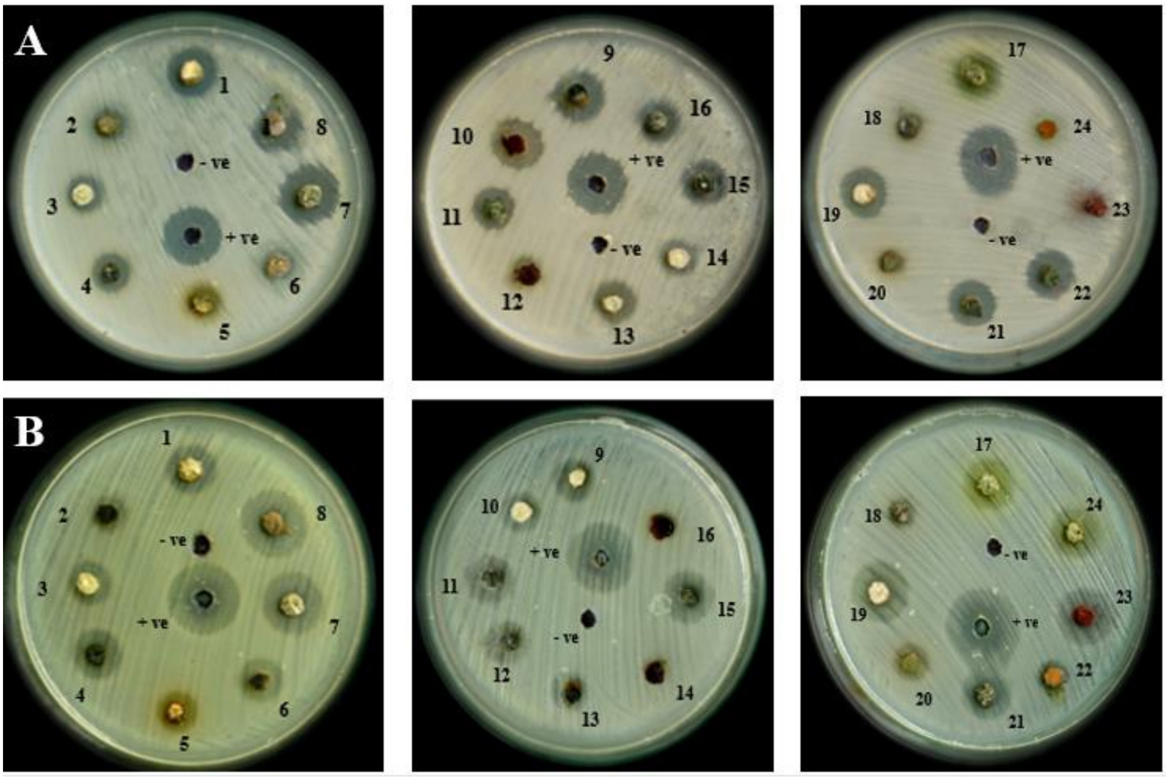

Figure S1. Screening of fungal discs (*Trichoderma* spp.) for antibacterial activity against *Xanthomonas oryzae* pv. *oryzae* A- MBXoo69, B- MBXoo53 (+- tetracycline - distilled water).

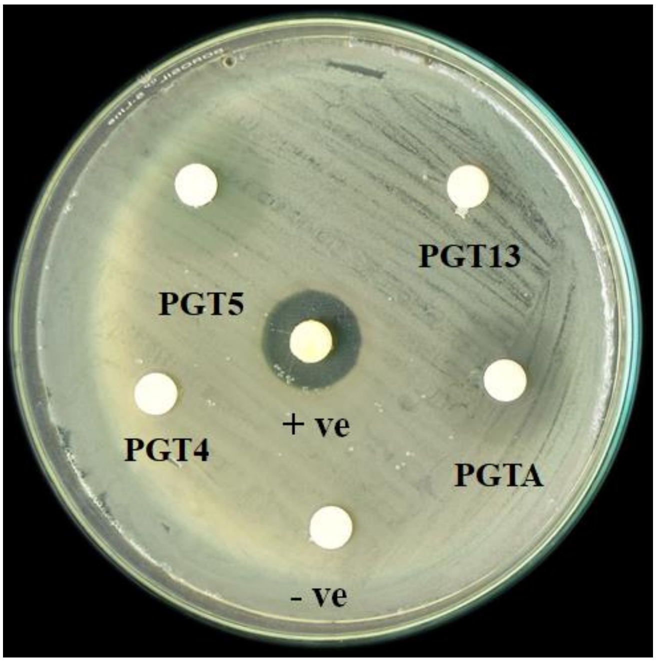

Figure S2: Antibacterial activity of zinc oxide nanoparticles (ZnO NPs) against *Xanthomonas oryzae* pv. *oryzae*(Xoo) by disc diffusion method (+ ve = tetracycline; - ve = distilled water).

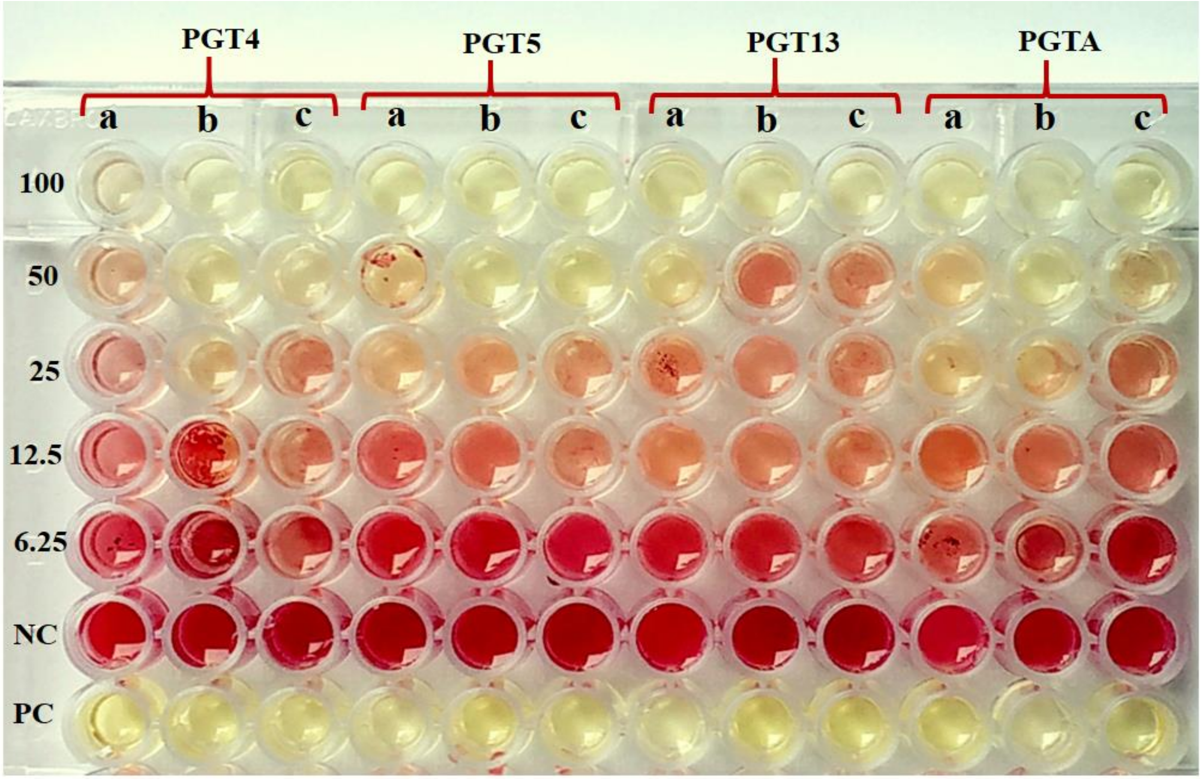

Figure S3: Bacterial sensitivity test of biosynthesized zinc oxide nanoparticles from *Trichoderma harzianum* (PGT4) (MH429899.1), *Trichoderma reesei* (PGT5)(MH429901.1), *Trichoderma reesei* (PGT13) (MH429900.1) and co-culture of *Trichoderma* sp. (PGTA) against different strains of plant pathogen *Xanthomonas oryzae* pv. *oryzae* (MF579736.1) (concentration in µg/ml) (a- Trail 1, b- Trail 2, and c- Trail 3).
